# Refugees, Forced Displacement, and War^1^

**DOI:** 10.3201/eid1011.040624_03

**Published:** 2004-11

**Authors:** Trude Bennett, Linda Bartlett, Oluwasayo Adewumi Olatunde, Lynn Amowitz

**Affiliations:** * University of North Carolina at Chapel Hill, Chapel Hill, North Carolina, USA;; † Centers for Disease Control and Prevention, Atlanta, Georgia, USA;; ‡ University of British Columbia, Vancouver, British Columbia, Canada;; § Physicians for Human Rights, Boston, Massachusetts, USA

**Keywords:** refugees, forced displacement, war

Women make up high proportions of refugee and internally displaced populations, and they suffer unique consequences of war and conflict because of gender-based violence, discrimination, and caretaking roles. Refugee women are especially vulnerable to infectious disease, as well as threats to their mental health and physical safety.

## Infectious Causes of Maternal Death in Refugee Populations in Afghanistan

The Reproductive Age Mortality Survey (RAMOS) in Afghanistan consisted of death identification followed by death investigation. The study identified 357 deaths of women of reproductive age (15–49 years) among residents of >16,000 Afghani households and investigated 80% of these deaths through the verbal autopsy method. The maternal death rate is extremely high (1,600–2,200 deaths per 100,000 live births) in Afghanistan as a whole, and the estimate in one study site was the highest ever recorded (6,500/100,000 live births in Ragh, Badakshan). The vast majority of maternal deaths were attributed to direct obstetric causes. Infectious causes, primarily tuberculosis, malaria, and postpartum sepsis, accounted for 12% of deaths. Tetanus, tuberculosis, and malaria often claimed women's lives while they were pregnant.

Women faced substantial barriers to care, and very few accessed preventive or curative services. In a country of very low resources and conflict such as Afghanistan, policy development and program implementation to reduce maternal deaths are challenging. Causes of maternal death are multifactorial and cannot be resolved simply by increasing the percentage of deliveries by skilled birth attendants. Infectious causes of death identified in this study illustrate the need for comprehensive maternity care, including preconceptional, prenatal, and postnatal care, integrated with other reproductive health and primary care services.

## Impact of War on Women's Health: Refugees from Liberia and Sierra Leone in Nigeria

A study carried out between January and March 2004 with Liberian refugee women residing in the United Nations refugee camp at Oru village in Ogun State, Nigeria, shows how forced migration contributes to increased incidence of both communicable and noncommunicable diseases in women. Liberia's civil war resulted in approximately 215,000 refugees at the end of 2001; 50% to 80% of these refugees were women. During the civil war, an estimated 40% of all Liberian women were raped. Loss of family forces women to depend on men and may lead to rape, forced marriage, prostitution, domestic abuse, and increasing risk of HIV and other sexually transmitted infections. Lack of postwar shelter compounds other problems and increases exposure to mosquitoborne diseases. Lack of clean drinking water introduces risks of bacillary dysentery, cholera, diarrheal disease, typhoid, hepatitis A, and other diseases.

Researchers concluded that solutions to the negative impact of war on women's health should be based in education, empowerment, efficient publicity, and effective policies. A sub-ministry devoted to women's affairs and maternal and child health was recommended, with funding specifically earmarked for women's health. Regular screening for preventable or treatable disease should be done in the home country and continued after the safety period ends.

## Violations of International Women's Rights: Effects on the Overall Health of Women

Findings from a study by Physicians for Human Rights indicate that nearly half of all households in three southern cities in Iraq experienced human rights abuses among household members between 1991 and 2003. Such abuses represent considerable challenges for justice and accountability and emphasize the need to address individual and community mental health needs on a large scale. The prevalence of mental illness represents a challenge to the Iraqi health system, since <100 psychiatrists are reported to practice in the country, and therapeutic medications and social support systems are lacking.

Households surveyed expressed support for a government that would protect and promote human rights, including the rights of women. However, the lack of support for certain women's rights by both men and women may make the full range of women's human rights difficult to achieve. Consequently, restrictions on women's rights or ineffective representation of women may have substantial, adverse health consequences for women and girls. This study suggests the need for a gender- and rights-based approach for reconstruction and community health and development in Iraq.

